# Activity-induced spontaneous spikes in GABAergic neurons suppress seizure discharges: an implication of computational modeling

**DOI:** 10.18632/oncotarget.15660

**Published:** 2017-02-23

**Authors:** Wei Lu, Jing Feng, Bo Wen, Kewei Wang, Jin-Hui Wang

**Affiliations:** ^1^ Qingdao University, School of Pharmacy, Qingdao, Shandong, China; ^2^ State Key lab for Brain and Cognitive Sciences, Institute of Biophysics, Chinese Academy of Sciences, Beijing, China; ^3^ University of Chinese Academy of Sciences, Beijing, China

**Keywords:** Epilepsy, spikes, neuron, synaptic transmission and GABA, Pathology Section

## Abstract

**Background:**

Epilepsy, a prevalent neurological disorder, appears self-termination. The endogenous mechanism for seizure self-termination remains to be addressed in order to develop new strategies for epilepsy treatment. We aim to examine the role of activity-induced spontaneous spikes at GABAergic neurons as an endogenous mechanism in the seizure self-termination.

**Methods and Results:**

Neuronal spikes were induced by depolarization pulses at cortical GABAergic neurons from temporal lobe epilepsy patients and mice, in which some of these neurons fired activity-induced spontaneous spikes. Neural networks including excitatory and inhibitory neurons were computationally constructed, and their functional properties were based on our studies from whole-cell recordings. With the changes in the portion and excitability of inhibitory neurons that generated activity-induced spontaneous spike, the efficacies to suppress synchronous seizure activity were analyzed, such as its onset time, decay slope and spike frequency. The increases in the proportion and excitability of inhibitory neurons that generated activity-induced spontaneous spikes effectively suppressed seizure activity in neural networks. These factors synergistically strengthened the efficacy of seizure activity suppression.

**Conclusion:**

Our study supports a notion that activity-induced spontaneous spikes in GABAergic neurons may be an endogenous mechanism for seizure self-termination. A potential therapeutic strategy for epilepsy is to upregulate the cortical inhibitory neurons that generate activity-induced spontaneous spikes.

## INTRODUCTION

Cortical seizure onset results presumably from an imbalance of neuronal excitation and inhibition toward the synchronous overexcitation of network neurons [1–5]. After antiepileptic medications are given by strengthening GABAergic synapses and inhibiting neuronal spiking [6], some epileptic patients, especially temporal lobe epilepsy, become drug-resistant [7, 8]. Thus, comprehensive picture about pathological characteristics of seizure-onset neuronal networks in intractable epileptic patients remains to be elucidated. On the other hand, because seizure activity is an automatic termination, there may be endogenous mechanisms for seizure self-termination in the brain [9, 10], such as the functional upregulation of GABAergic neurons, to arrest epilepsy [10, 11].

In terms of a role of GABAergic neurons in seizure termination, a portion of these neurons with functional upregulation emerge in the temporal lobe cortices of temporal lobe epilepsy (TLE) patients, which facilitates seizure self-termination [11]. Intensive activities in GABAergic neurons induce their spontaneous action potentials, i.e., activity-induced spontaneous spikes (AISS) [12–14], especially in seizure-onset tissues [10]. A hypothesis is that overexcited neural networks trigger AISS generation in GABAergic neurons, which in turn suppresses the network overexcitation. There are two strategies address this hypothesis, i.e., molecular biology to upregulate AISS-generated GABAergic neurons in their portions, spiking abilities or both as well as computational modeling to simulate whether an upregulation of AISS-generated GABAergic cells arrests seizure activity. The computational modeling will quickly give us the guideline whether the portion and/or spiking capability of AISS-generated GABAergic neurons should be preferentially upregulated through molecular manipulations to arrest epilepsy.

The second strategy has been used in our study, in which the function upregulation of GABAergic neurons was analyzed in seizure-onset tissues from TLE patients and mice, and then the simulation about the role of AISS-generated inhibitory neurons as an endogenous mechanism in seizure self-termination was conducted based on the experimental data. This simulation is critically needed, since previous computation modeling has been focused to examine the mechanisms of the seizure onset, the balance between the excitatory and inhibitory neurons, and their interactions [15–20].

## RESULTS

### A portion of inhibitory neurons is upregulated for activity-induced spontaneous spikes in seizure-onset tissue

**Figure 1:**
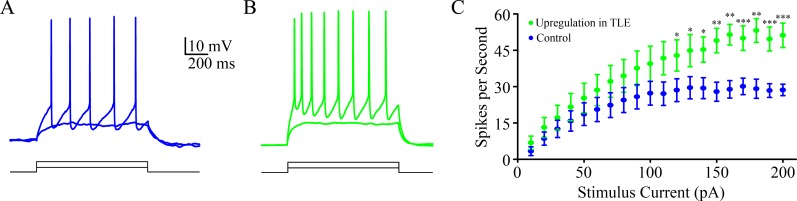
A portion of inhibitory neurons is functionally upregulated in seizure-onset tissues of TLP patients **A**. shows depolarization-induced spikes at an inhibitory neuron in temporal lobe. **B**. shows depolarization-induced spikes in an inhibitory neuron from tissue-onset tissue of TLE patient. **C**. shows input-output curves from these two groups of neurons. An asterisk denotes *p* < 0.05, two asterisks denote *p* < 0.01 and three asterisks denote *P*,0.001.

The functional states of inhibitory neurons in temporal lobe of TLE patients were assessed by whole-cell recording. Their input-output curves were analyzed by inducing action potentials with depolarization pulses. Certain inhibitory neurons appear to have a high ability to convert excitatory inputs into spikes, as showed in Figure 1A-1B. Figure 1C shows the input-output curves for the group of inhibitory neurons with high ability to produce the spikes (green symbols, *n* = 7 cells), compared with other neurons in this area (blues, *n* = 11 cells). This result suggests that a portion of inhibitory neurons in temporal lobe of TLE patients is functionally upregulated.

**Figure 2:**
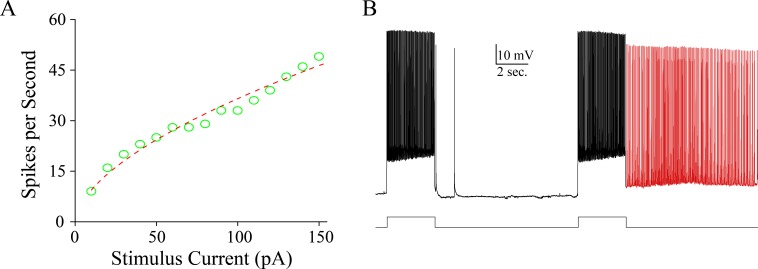
Intensive activity induces spontaneous spikes in inhibitory neurons form the seizure-onset tissues of TLP patients **A**. illustrates an input-output curve from an inhibitory neuron from seizure-onset tissue of TLE patient. **B**. shows that intensive activity induces spontaneous spikes in this inhibitory neuron. Calibration bars are 10 mV and 2 seconds.

**Figure 3:**
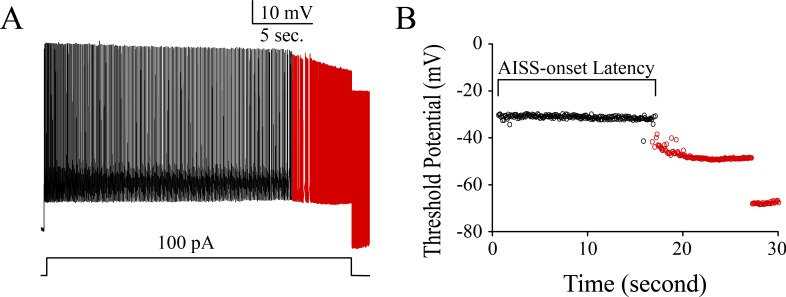
Intensive activity induces spontaneous spikes in cortical GABAergic neurons from the mice **A**. shows that intensive activity induces spontaneous spikes in a GABAergic neuron. Calibration bars are 10 mV and 5 seconds. **B**. illustrates the changes of threshold potentials during AISS induction and expression.

In analyzing the ability of firing activity-induced spontaneous spikes (AISS), we have found that intensive activity in the inhibitory neurons with high ability to produce spikes in temporal lobe of TLE patients can be easily induced to fire AISS (Figure 2). Their features are similar those in cortical GABAergic neurons in the mice (Figure 3 and [12]). Subsequently, we have examined the role of these AISS-generated neurons from seizure-onset tissues of TLE patients in seizure self-termination by the computational simulation.

### The principles used in computational modeling about AISS-generated neurons for seizure termination

The neuronal networks are computationally simulated based on our previous experimental data, which include glutamatergic excitatory neurons and GABAergic inhibitory neurons in the ratio of 4 to 1, which are synaptically connected [11]. 30% of GABAergic neurons are AISS-generated neurons [12]. Based on the properties of AISS-generated inhibitory neurons, such as their portions, spiking frequency and AISS-onset latency (Figure 3), we change these parameters and examine their influence on efficacies of seizure suppression in neuronal networks.

The output activity of excitatory neurons is digitized as spike frequency (calibration bar in Supplementary Figure S4). Without exogenous stimulation to the excitatory neurons, their spiking frequencies from low to high are thought to be their spontaneous activity. Their intensive and synchronous spikes are thought to be seizure activity, or vice versa as seizure termination. The activity levels of these excitatory neurons are quantified as the strength (frequency) and the duration of seizure spikes (Supplementary Figure S4). The neuronal network with persistent and high frequency spikes is called as seizure networks, compared with the silence network as normal network. The efficacy of seizure suppression is merited based on the onset time, decay slope and minimal spiking frequency of seizure termination.

### An upregulation in the portion of AISS-generated inhibitory neurons leads to seizure termination

**Figure 4:**
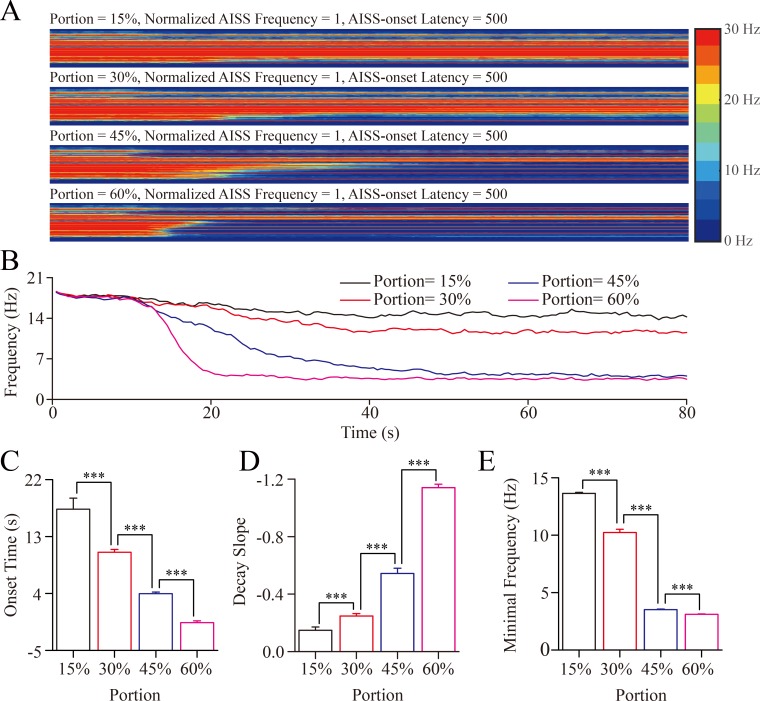
The upregulation in the portion of AISS-generated inhibitory cells leads to seizure termination A. By increasing the portion of AISS-generated inhibitory neurons under the conditions of their spiking frequency normalized at 1 and AISS-onset latency at 500-induced spikes, the strengths of seizure spikes and their spike frequency appear reduced **B**. shows the averaged strength of seizure activity *versus* time under the conditions of AISS-generated inhibitory neuron portions at 15%, 30% 45% and 60%. When the portions of AISS-generated neurons increase from 15% to 60%, the onset time of seizure suppression is progressively shortened, the decay slope of seizure suppression is increased, and the minimal spike frequency is decreased. **C**. The average values for the onset-time of seizure suppression are shortened when the portions of AISS-generated neurons increase. **D**. The decay slopes of seizure strength rise when the portions of AISS-generated neurons increase. **E**. The minimal fire frequency of the network decreases when the proportions of AISS-generated neurons increase.

We change the portion of AISS-generated inhibitory neurons under the conditions of their spiking frequency normalized at 1 and AISS-onset latency at 500-induced spikes. With the increase in the portion of AISS-generated inhibitory neurons, the number of excitatory neurons that fire synchronous spikes and their spike frequency appear reduced (Figure 4A). When the portions of AISS-generated neurons are increased from 15% to 60%, the onset time of seizure suppression is progressively shortened, the decay slope of seizure suppression is raised, and the minimal spike frequency at the excitatory neurons is decreased (Figure 4B).

The values for the onset-time of seizure suppression are 17.32±1.75, 10.53±0.45, 3.95±0.28 and -0.61±0.27 seconds correspondent to the portions of AISS-generated GABAergic neurons in total inhibitory neurons at 15%, 30%, 45% and 60%, respectively (Figure 4C; *p* < 0.001, one-way ANOVA). The values in the decay slope of seizure suppression are -0.15±0.02, -0.25±0.2, -0.54±0.04 and -1.14±0.02 correspondent to the portions of AISS-generated inhibitory cells at 15%, 30%, 45% and 60% (Figure 4D; *p* < 0.001, one-way ANOVA). The values in minimal spike frequency are 13.63±0.1, 10.22±0.3, 3.5±0.05 and 3.11±0.03 Hz correspondent to the portions of AISS-generated inhibitory neurons at 15%, 30%, 45% and 60% (Figure 4E; *p* < 0.001, one-way ANOVA). These results indicate that the increased portion of AISS-generated inhibitory neurons can effectively attenuate seizure spikes.

### The upregulated spike frequency of AISS-generated inhibitory cells facilitates seizure termination

**Figure 5:**
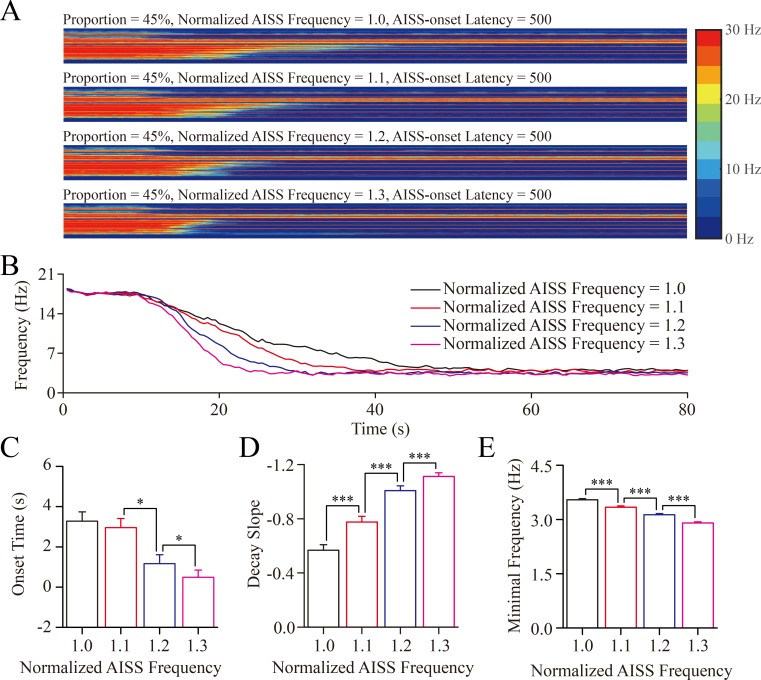
The upregulation in the spike frequency of AISS-generated inhibitory cells facilitates seizure termination A. By increasing the spike frequency of AISS-generated inhibitory neurons under the conditions of their portion at 45% and AISS-onset latency at 500-induced spikes, the strengths of seizure spikes and their spike frequency appear reduced **B**. shows the averaged strength of seizure activity *versus* time under the conditions of normalized fire frequency at 1, 1.1, 1.2 and 1.3. When the normalized fire frequency of AISS-generated neurons increases from 1 to 1.3, the onset time of seizure suppression is progressively shortened, the decay slope of seizure suppression is increased, and the minimal spike frequency is decreased. **C**. The average values for the onset-time of seizure suppression are shortened when the spike frequencies of AISS-generated neurons increase. **D**. The decay slopes of seizure strength rise when the spike frequencies of AISS-generated neurons increase. **E**. The minimal fire frequency of the network decreases when the spike frequencies of AISS-generated neurons increase.

The spike frequencies of AISS-generated inhibitory neurons are changed under the conditions of their portion at 45% and AISS-onset latency at 500-induced spikes. When the normalized spike frequency at AISS-generated inhibitory neurons is increased from 1.0 to 1.3, the onset time of seizure suppression is progressively shortened, the decay slope of seizure suppression becomes increased, and the minimal spike frequencies at the excitatory neurons are decreased (Figure 5A-5B).

The values for the onset-time of seizure suppression are 3.28±0.46, 2.95±0.45, 1.17±0.44 and 0.49±0.36 seconds correspondent to the spike frequency of AISS-generated inhibitory neurons at 1, 1.1, 1.2 and 1.3, respectively (Figure 5C; *p* < 0.05, one-way ANOVA). The values in the decay slope of seizure suppression are -0.57±0.04, -0.77±0.04, -1.01±0.03 and -1.11±0.03 correspondent to the spike frequency of AISS-generated inhibitory neurons at 1, 1.1, 1.2 and 1.3, respectively (Figure 5D; *p* < 0.001, one-way ANOVA). The values in minimal spike frequency are 3.54±0.03, 3.34±0.04, 3.13±0.03 and 2.90±0.03 Hz, correspondent to the spike frequency of AISS-generated inhibitory cells at 1, 1.1, 1.2 and 1.3, respectively (Figure 4E; *p* < 0.001, one-way ANOVA). These data indicate that an increased spike frequency at AISS-generated inhibitory neurons can effectively attenuate seizure spikes.

### The upregulation of AISS-onset latency may not effectively lead to seizure termination

**Figure 6:**
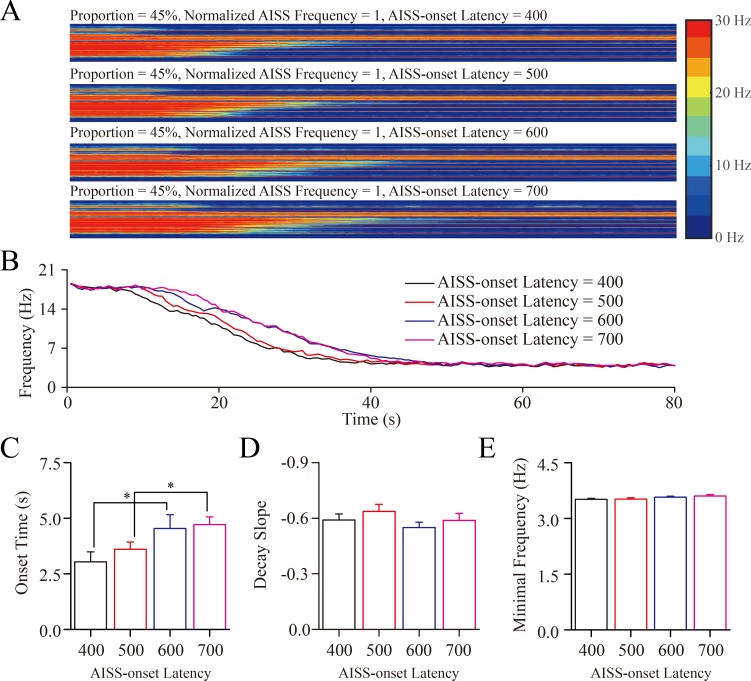
The AISS-onset latency did not significantly affect seizure termination **A**. By increasing the AISS-onset latency of AISS-generated inhibitory neurons under the conditions of their portion at 45% and spiking frequency normalized at 1, the strengths of seizure spikes and their spike frequency appear not reduced. **B**. shows the averaged strength of seizure activity *versus* time under the conditions of AISS-onset latency at 400, 500, 600 and 700 spikes. When the AISS-onset latency of AISS-generated neurons increases from 400 to 700, the onset time of seizure suppression is progressively shortened, but the decay slope of seizure suppression and the minimal spike frequencies are not changed. **C**. The average values for the onset-time of seizure suppression are prolonged when the AISS-onset latency of AISS-generated neurons increases. **D**. The decay slopes of seizure strength do not change when the AISS-onset latency of AISS-generated neurons increases. **E**. The minimal fire frequencies of the network do not change when the AISS-onset latency of AISS-generated neurons increases.

AISS-onset latencies are changed under the conditions of the portion of AISS-generated neurons at 45% and their spiking frequency normalized at 1. Although these settings suppress seizure spikes, the increased AISS-onset latency appears not obviously to inhibit synchronous spikes at excitatory neurons (Figure 6A-6B). The averaged values for the onset-time of seizure suppression are 3.04±0.45, 3.61±0.32, 4.54±0.62 and 4.72±0.35 seconds correspondent to AISS-onset latencies at the 400, 500, 600 and 700-induced spikes, respectively (Figure 6C; *p* = 0.028, one-way ANOVA, one asterisk shows *p* < 0.05). The values for the decay slope of seizure suppression are -0.59±0.03, -0.64±0.04, -0.55±0.03 and -0.59±0.04 correspondent to AISS-onset latencies at 400, 500, 600 and 700-induced spikes, respectively (Figure 6D; *p* = 0.369, one-way ANOVA). The values in minimal spike frequency are 3.51±0.03, 3.52±0.04, 3.57±0.03 and 3.61±0.03 Hz correspondent to AISS-onset latencies at 400, 500, 600 and 700 spikes, respectively (Figure 6E; *p* = 0.132, one-way ANOVA). These results indicate that AISS-onset latencies at GABAergic neurons may not effectively attenuate seizure spikes.

### The combination of AISS-neuron portion and frequency facilitates seizure termination

To reveal the coordinated effects of the portion, spike frequency and AISS-onset latency of AISS-generated inhibitory neurons on seizure suppression, we introduced the pairs of these parameters into computation-simulated neuronal networks. Stimulations of each condition were processed 30 times before two-way ANOVA analyses were conducted.

**Figure 7:**
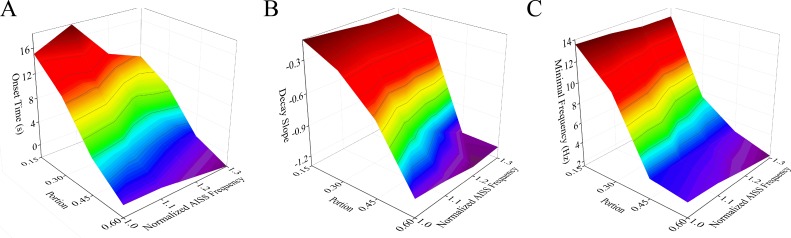
The portion and spike frequency of AISS-generated cells have a synergistic effect on seizure suppression **A**. The average values for the onset-time of seizure suppression are shortened when the portion and spike frequency of AISS-generated neurons increase. **B**. The decay slopes of seizure strength rise when the portion and spike frequency of AISS-generated neurons increase. **C**. The minimal fire frequency of the network decreases when the portion and spike frequency of AISS-generated neurons increase.

Figure 7 illustrates that the changes in both the portion of AISS-generated inhibitory neurons and their spike frequency can shorten the onset time, raise the decay slope and lower the minimal frequency of seizure spikes at the excitatory neurons. Two-way ANOVA analyses indicate that the increased portions and spike frequencies of AISS-generated neurons significantly suppress seizure spikes, compared with their individual action. Thus, the portion and spike frequency of AISS generated cells have a synergistic effect on seizure suppression.

**Figure 8:**
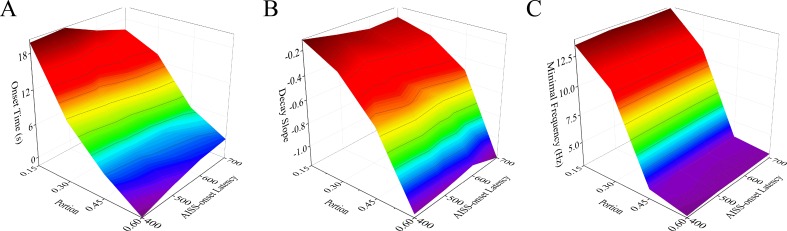
The portion and AISS-onset latency of AISS generated cells have no synergistic effect on seizure suppression **A**. The average values for the onset-time of seizure suppression are shortened when the portion and AISS-onset latency of AISS-generated neurons increase. Two-way ANOVA analysis indicates the portion and AISS-onset latency have no additive effect on the onset-time of seizure suppression. **B**. The decay slopes of seizure strength rise when the portion and AISS-onset latency of AISS-generated neurons increase. Two-way ANOVA analysis indicates the portion and AISS-onset latency have no additive effect on the decay slope of seizure suppression. **C**. The minimal fire frequency of the network decreases when the portion and AISS-onset latency of AISS-generated neurons increase. Two-way ANOVA analysis indicates the portion and AISS-onset latency have no additive effect on the minimal fire frequency of the network.

Figure 8 illustrates that the changes in both the portion of AISS-generated inhibitory neurons and AISS-onset latency can shorten the onset time, raise the decay slope and lower the minimal frequency of seizure spikes at the excitatory neurons. Two-way ANOVA analyses indicate that the increased portion and the decreased AISS-onset latency of AISS-generated inhibitory neurons has no additive effect seizure suppression, compared with their individual action. In other words, the portion and the AISS-onset latency of the AISS generated inhibitory cells do not have synergistic effect on seizure suppression.

**Figure 9:**
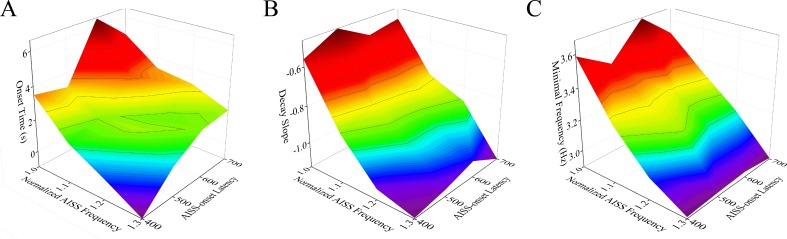
The spike frequency and AISS-onset latency of AISS-generated cells have no synergistic effect on seizure suppression **A**. The average values for the onset-time of seizure suppression are shortened when the spike frequency and AISS-onset latency of AISS-generated neurons increase. Two-way ANOVA analysis indicates the portion and AISS-onset latency have no additive effect on the onset-time of seizure suppression. **B**. The decay slopes of seizure strength rise when the spike frequency and AISS-onset latency of AISS-generated neurons increase. Two-way ANOVA analysis indicates the portion and AISS-onset latency have no additive effect on the decay slope of seizure suppression. **C**. The minimal fire frequency of the network decreases when the spike frequency and AISS-onset latency of AISS-generated neurons increase. Two-way ANOVA analysis indicates the portion and AISS-onset latency have no additive effect on the minimal fire frequency of the network.

Figure 9 shows that the spike frequency at AISS-generated inhibitory cells and their AISS-onset latency can shorten the onset time, raise the decay slope and lower the minimal frequency of synchronous spikes at excitatory neurons. Two-way ANOVA analyses indicate that the spike frequency and AISS-onset latency of AISS-generated inhibitory neurons has no additive effect seizure suppression, compared to their individual action. Thus, the AISS-onset latency and spike frequency of AISS generated inhibitory cells do not have synergistic effect on seizure suppression.

## DISCUSSION

Our study indicates that a portion of cortical inhibitory neurons in seizure-onset tissues from TLE patients is functionally upregulated to easily fire activity-induced spontaneous spikes (Figures 1~3). By using computational simulation, we demonstrate that the upregulations in the portion of AISS-generated inhibitory neurons or their spike frequency significantly suppress synchronous spikes at the excitatory neurons of seizure neural network in terms of the onset time, decay slope and minimal frequency of seizure termination (Figures 4~5). The upregulated portion and spike frequency of AISS-generated neurons synergistically shorten the onset time, accelerate the decay and lower the minimal frequency of synchronous spikes at the excitatory neurons for seizure suppression (Figures 7~9). These results provide the clues for epileptic therapeutic strategy by upregulating AISS-generated neurons and their spike frequency with molecular biology approaches, which is being studied.

Computational simulation based on our experiment data indicates that the increases in the portion of AISS-generated GABAergic neurons and their spiking strengths reinforce cortical inhibitory networks to terminate seizure discharge. Inhibitory neuron-based therapy for epilepsy has been proposed [21]. The synergistic influences of upregulating AISS-generated inhibitory neurons and their spike frequency as well as downregulating AISS-onset latency on seizure suppression suggest that the epilepsy therapy will be benefit from strengthening the function of multiple subcellular compartments in AISS-generated inhibitory cells, such as their intrinsic properties and GABAergic output. In these regards, therapeutic strategies for intractable epileptic patients should be to increase both the number and function of AISS-generated inhibitory neurons.

Two strategies can be used to upregulate the portion and spiking capability of AISS-generated GABAergic neurons, optogenetic approach and stem cell implantation, which we currently pursue. Our previous study indicates the activation of voltage-independent sodium channels for the generation of activity-induced spontaneous spike [12]. The genes of channel-rhodopsin are being introduced into the AISS generated GABAergic neurons, and the activation of AISS-generated neurons by laser-induced channel-rhodopsin will be conducted *in vivo* to test our data from computational simulation, i.e., AISS-generated GABAergic neurons form seizure self-termination. On the other hand, stem cell therapy may be an option to raise the portion of AISS-generated inhibitory neurons as stem cells are preferentially differentiated to inhibitory neurons. The control of these stem cells being differentiated into AISS-generated inhibitory neurons is challenging since the differentiation of these stem cells into various types of inhibitory neurons is random. Therefore, the molecular mechanisms underlying the upregulations in the portion and function of AISS-generated inhibitory neurons remain to be addressed, as well as the approaches with molecular biology for specifically enhancing inhibitory neuron function and their differentiation from the stem cells remains to be developed

It is noteworthy that the uses of these two strategies to increase the portion and function of AISS-generated GABAergic neurons for seizure self-termination may lead to the imbalance and incoordination between excitatory and inhibitory neurons in the neural network for cognitive processes. In this regard, the arresting of seizure and the maintenance of healthy cognitive processes should be considered in future preclinical and clinical trials.

Computational simulations for seizure onset based on the studies in epileptic animal models have been done by changing one of the following factors, such as voltage-gated ion channels [20], local neural networks [18, 19], small-world versus large-scale network [16, 17] or destabilization for multi-state network transition [15]. Computational simulation in our study is focused on seizure self-termination based on our data from TLE patients versus control by introducing multiple factors (such as AISS-generated cell portion and spiking ability) into neural networks. In addition to a new direction for seizure self-termination, the results read from our simulations under the conditions of specific-type neurons and synapses make computational neural networks to be closely comprehensive.

## MATERIALS AND METHODS

### Ethic issues

The uses of brain tissues from the patients and the procedures of surgically dissecting their brain tissues have been approved by Ethic Committee of Human Tissue Use in General Hospital of Chinese Military (2010824001). The judgment for intractable neocortical TLE patients to receive a neurosurgical therapy was based on international criteria, e.g., resistant to the medications [22, 23]. The anesthetic and neurosurgical procedures used in the TLE patients were based on the standards approved by the Food and Drug Agency China. The approaches for cutting cortical slices and doing electrophysiological study were approved by the Institutional Animal Care and Use Committee in the Administration Office of Laboratory Animals in Beijing China (B10831).

### Brain tissues and slices

The blocks of temporal lobe cortices (1×1×1 cm) were harvested from the TLE patients. All of these neocortical TLE patients had been diagnosed as intractable drug-resistance. The seizure-onset cortices were localized by EEG showing epileptic and interictal discharges. After the skull was opened, the seizure cortices were identified by embedding 32-channels’ EEG in diagnosis-located areas [11], in which the areas with frequent interictal discharges and the earliest seizure onsets were defined as the seizure-onset cortices. In surgical operations, surgeons removed the seizure-onset parts of temporal lobe cortices. The tissues (1×1×1 cm) isolated from the assigned cortices *in vivo* were immediately cut into the blocks (1×1×0.6 and 1×1×0.4) in the modified and oxygenized (95% O_2_/5% CO_2_) artificial cerebrospinal fluid (ACSF, mM: 124 NaCl, 3 KCl, 1.2 NaH_2_PO_4_, 26 NaHCO_3_, 0.5 CaCl_2_, 5 MgSO_4_, 20 dextrose and 5 HEPES; pH 7.35) at 4°C. These blocks in this 4°C ACSF incubation were transferred to research laboratory for electrophysiological studies [11].

The slices (300 μm) from seizure-onset cortices in TLE patientss were cut by Vibratome in the modified and oxygenized ACSF (mM: 124 NaCl, 3 KCl, 1.2 NaH_2_PO_4_, 26 NaHCO_3_, 0.5 CaCl_2_, 5 MgSO_4_, 20 dextrose and 5 HEPES; pH 7.35) at 4°C, and were held in the normal oxygenated ACSF (mM: 126 NaCl, 2.5 KCl, 1.2 NaH_2_PO_4_, 26 NaHCO_3_, 2.0 CaCl_2_, 2.0 MgSO_4_, 10 dextrose and 5 HEPES; pH 7.35) 35°C for 1 hour before the experiments. These slices then were transferred to a submersion chamber that was perfused with normal ACSF for electrophysiological experiments [24–26].

### Electrophysiological studies in cortical interneurons

The selection of cortical interneurons for whole-cell recording was based on the following criteria. These neurons in layers II-IV of human cortices showed smaller round soma and multiple processes [11], compared to relatively larger pyramidal neurons, under the DIC microscope (Nikon, FN-E600). These interneurons demonstrated fast spiking, no adaptation in the spike amplitudes and frequencies as well as high magnitudes in after-hyperpolarization [11], typical properties for inhibitory interneurons [12, 27–29]. These interneurons were recorded by an amplifier (MultiClapm-700B, Axon Instrument Inc, CA USA) under whole-cell current-clamp. Electrical signals were inputted into pClamp-10 (Axon Instrument Inc.) with 20 kHz of sampling rate. The functions of inhibitory neurons were evaluated based on their intrinsic properties (such as input-output curve and spiking ability distribution). Pipette solution for recording action potentials included (mM) 150 K-gluconate, 5 NaCl, 0.4 EGTA, 4 Mg-ATP, 0.5 Tris- GTP, 4 Na-phosphocreatine and 5 HEPES (pH 7.4 adjusted by 2M KOH). The osmolarity of pipette solutions made freshly was 295-305 mOsmol, and the pipette resistance was 8-10 MΩ [30, 31].

In the analyses of input-output and membrane I-V curves [32], depolarization pulses (1 sec.) in various intensities were injected into these interneurons to induce the sequential spikes. In evaluating their ability of firing spikes, we measured the initiation of spikes by weak depolarization that was thought as neuronal sensitivity to excitatory inputs, i.e., neuronal ability to convert synaptic signals into spikes [33–36].

The data were analyzed if the recorded neurons had resting membrane potentials negatively more than -60 mV and action potentials above 90 mV. The criteria for the acceptation of each experiment also included less than 5% changes in resting membrane potential, spike magnitudes, input and seal resistances throughout each experiment. The values of neuronal input-outputs are presented as mean±SE. The comparisons between groups are done by one-way ANOVA [37–39].

### Modeling of individual neurons

The computational simulation of each neuron was based on a regular leaky integrate-and-fire neuron model with a stochastic component [16, 40]. The formula was given by

$$\frac{dV(t)}{dt}=\frac{I(t)}{C_{m}}-\frac{V_{m}-V_{\text{rest}}}{R_{m}C_{m}}+\xi (t)$$ (1)

V_*m*_ is membrane potential, V_*7*_ is resting membrane potential, I is input current, C_*m*_ is membrane capacitance, R_*m*_ is membrane resistance, and ξ is stochastic component (white noise). These neurons fired spikes when V_*m*_ reached a threshold (V_*th*_) triggered by input currents, which were injected into the neurons. When each neuron fires spikes, its membrane reversal potential was set at 30 mV. Neuronal membrane potential then returned to the resting membrane potential. These neurons were able to fire spikes after the end of absolute refractory period (ARP). The values of these parameters were set based on our previous data in Table 1 [11]. Matlab (MathWorks, Natick, MA) was used to construct model neurons. A simulated spike in an individual neuron was shown in Supplementary Figure S1.

**Table 1:** Physiological properties for different types of neurons

Physiological properties	Excitatory neurons	GABAergic neurons
Rest membrane potential, V_*rest*_ (mV)	−69.6±4.6	−60.5±4.2
AP threshold potentials, V_*th*_ (mV)	−41.7±0.12	−38.6±0.1
Membrane capacitance, C_*m*_ (pF)	82±24.4	77.6±25.8
Membrane resistance, R_*m*_ (MΩ)	291±76.7	305±50.8
Membrane potential, V_*m*_ (mV)	−69.6±0.46	−60.5±0.42
Refractory period, RP (ms)	30.2±3.10	9.8±1.4

### Synaptic inputs

In terms of the role of ligand-gated ion channels in synaptic activities, AMPAR and NMDAR were assigned to mediate excitatory postsynaptic potentials, and GABA_A_R were assigned to mediate inhibitory postsynaptic potentials. For the postsynaptic receptor conductance, the waveform was specified by the difference of two functional exponentials [41–44]:

$$g=\bar{g}\left(e^{\frac{-t}{\tau_{2}}}-e^{\frac{-t}{\tau_{1}}}\right)$$ (2)

τ_1_ is activation time constant, τ_2_ is decay time constant, and $\bar{g}$ is a scaled factor. The synaptic input is then specified by Ohm's formula:

$$I=g(V_{m}-E_{\text{syn}})$$ (3)

V_*m*_ is membrane potential, E_*syn*_ is a reversal potential of synaptic current, *g* is receptor conductance.

The values of these parameters in Table 2 for three types of synaptic currents were based on the data measured by voltage-clamp [45, 45–53]. The simulations of single receptor current in voltage-clamp were shown in Supplementary Figure S2, where the membrane potentials were held at -40mV for GABA_A_R response and -70mV for AMPAR and NMDAR responses.

**Table 2:** Functional dynamics of ligand-gated receptor channels in the synapses

	AMPA receptors	NMDA receptors	GABA receptors
g (nS)	1	0.1	0.6
τ_1_ (ms)	0.5	5	0.5
τ_2_ (ms)	2	150	7
E_*syn*_ (mV)	0	0	−70

### Network and synaptic connections

The principle to build the neuronal network in computational modeling was described below. The simulated neuronal network in cerebral cortex includes excitatory and inhibitory neurons, in which their ratios were 80% *versus* 20% [54]. These excitatory neurons and inhibitory interneurons were synaptically connected to form input and recurrent excitations as well as feedforward and feedback inhibitions (Supplementary Figure S3). The inhibitory neurons by their axons innervated the excitatory neurons to coordinate their activities [28, 55]. The excitatory neurons projected to other regions through their long axons, i.e., small-world model [16]. The weight of synaptic connections on excitatory and inhibitory neurons in the simulated networks was presumably similar. All neurons in our simulated network were connected by excitatory and inhibitory synapses (Supplementary Figure S3). Based on such principles, we built the simplified network for seizure-onset and seizure-termination. The neurons in the simulated network were thought as integrate-fire cell model [56–58]. Their functional status introduced into the simulated networks was digitized from our data in Tables 1–3. Inhibitory neurons in the networks were divided into two groups, i.e., AISS-generated GABAergic cells and non-AISS GABAergic cells.

**Table 3:** Parameters and their values used in network construction

Parameter	Values
Number of neurons in network	100
Rate of GABAergic neurons	20%
Connections to neighboring neurons per neuron	8 for excitatory neurons
	24 for inhibitory neurons
Sampling Rate	1500 per second
Simulation time	80s
AISS-generated neurons in total GABAergic neurons	Variable from 15% to 60%
Ratio of maximal fire frequency of AISS-generated neurons in the test network to that in the origin network	Variable from 1 to 1.3
Spikes needed to trigger AISS	Variable from 400 to 500

To more easily constrain activity to spread in a controlled manner, and to eliminate the effects of boundary conditions, we restricted our analyses to a “rings” of neurons. One hundreds of neurons were placed in the simulated network, in which eighty of the neurons were excitatory neurons and twenty were inhibitory neurons [16]. The two types of neurons were evenly distributed in the neural network. Each neuron was connected to other neurons in which an excitatory connected to eight neighboring neurons and an inhibitory neuron connected twenty-four neighboring neurons. The values of their physiological properties were given in Supplementary Table S1. Postsynaptic receptors were NMDA and AMPA if the synapses received glutamatergic axon inputs, and postsynaptic receptors were GABA_A_ if the synapses received GABAergic axon inputs. An illustration of neuronal networks with varying amount of neurons and connections was presented in Supplementary Figure S3. These parameters and their values used in the simulated neural network construction were shown in Table 3.

Synaptic activities at each neuron are generated by using Matlab and distributed by a Poisson distribution:

$$P(X=k)=\frac{\lambda^{k}}{k!}e^{-\lambda }$$ (4)

λ indicates the average number of synaptic events per time interval. With one second interval, λ values are 2.86 for AMPA and NMDA receptors and 0.91 for GABA receptor, based on our previous data [11]. To induce seizure discharges, AMPA and NMDA currents were upregulated about 30%, while GABA currents were downregulated about 30% manually. The upregulation of excitatory synapses and downregulation of inhibitory synapses induced abrupt and synchronous seizure activities in the computational-simulated networks (Supplementary Figure S4).

### AISS-generated neurons and their placement into the simulated network

AISS-generated GABAergic inhibitory neurons were introduced in seizure network that fired simultaneous and synchronous spikes. To test the role of AISS-generated neurons in seizure termination, we manipulated the following parameters. The portion was defined as a ratio of AISS-generated inhibitory neurons to non-AISS inhibitory neurons, whose default value was set at 30%. The AISS-generated inhibitory neurons were evenly distributed in the simulated network. AISS-onset latency was merited by time period for certain number of induced spikes to trigger AISS (Supplementary Figure S3), whose default value was set as 500-induced spikes. As a portion of GABAergic neurons was functionally upregulated in seizure-onset cortices (Supplementary Figure S4C-S4D), the ratio of maximal firing frequency in epilepsy network to that in normal network, named as “Normalized AISS Frequency”, was introduced into the network with a default value at one. The duration of AISS firing was 60 seconds. These default values were given based on experimental data [12–14, 59]. Furthermore, the values of these parameters were changed (also see Table 3). For single-variation analyses, the simulations were performed 40 times for each condition. For dual-variation analyses to reveal the synergistic effects, the simulations were performed 30 times for each condition.

### Data analysis

The computational simulations and data analyses were conducted by using Matlab. Action potentials were assumed to be detected if their potential changes reach to 30 mV. The time of each detected spike was measured. The averaged spike frequency at each excitatory neuron was measured by a time window of 500 milliseconds. The strength of seizure activity was presented as the mean value of spike frequency at the excitatory neurons *versus* time (Supplementary Figure S4C). Three functions were measured to quantify the role of AISS in the seizure suppression. Onset time for seizure suppression was the time from AISS onset to the time when the seizure strength declined to 80% of its origin values. Decay slope was the decay slope of seizure suppression from 80% to 20%. Minimal frequency was AISS-suppressed synchronous spikes in the seizure network. Single-variation simulation data was analyzed using one-way ANOVA, and dual-variation simulation data was analyzed using two-way ANOVA. *t*-test was used for the data comparison from two groups.

## SUPPLEMENTARY MATERIALS FIGURES AND TABLE




