# Supplementary material for: Activity-induced spontaneous spikes in GABAergic neurons suppress seizure discharges: an implication of computational modeling
Source: Oncotarget. 2017 Feb 23;8(20):32384–97. doi: 10.18632/oncotarget.15660 (PMC5464796; doi:10.18632/oncotarget.15660)
Supplement: Supplementary file 1 [file oncotarget-08-32384-s001.pdf]

Activity-induced spontaneous spikes in GABAergic neurons suppress seizure discharges: an implication of computational modeling

Supplementary Material

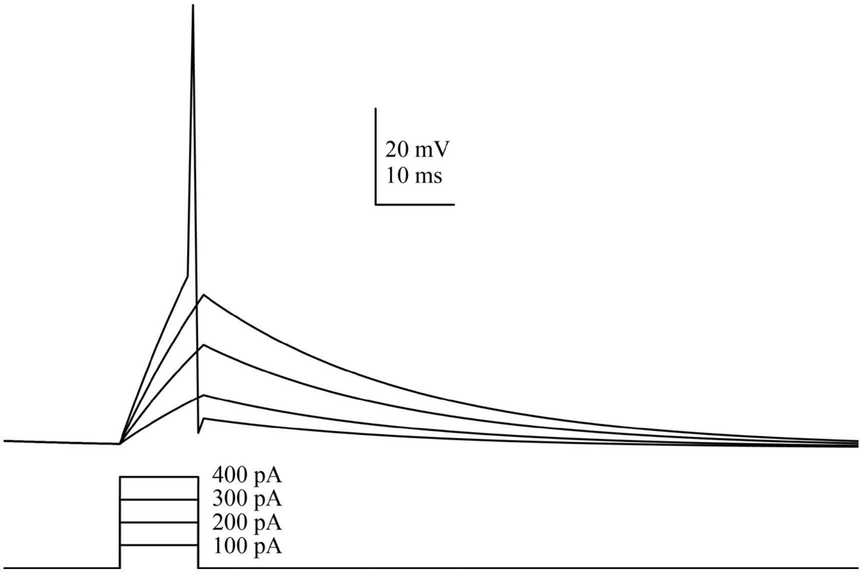

Supplementary Figure S1: the membrane potential of an integrate-and-fire neuron model under different input currents. Input currents of 100 pA to 400 pA are applied to an integrate-and-fire neuron model with the parameters: Vrest = -70 mV, Vth = -40 mV, Cm = 103.04 pF, Rm = 315.45 MΩ, RP = 28 ms.

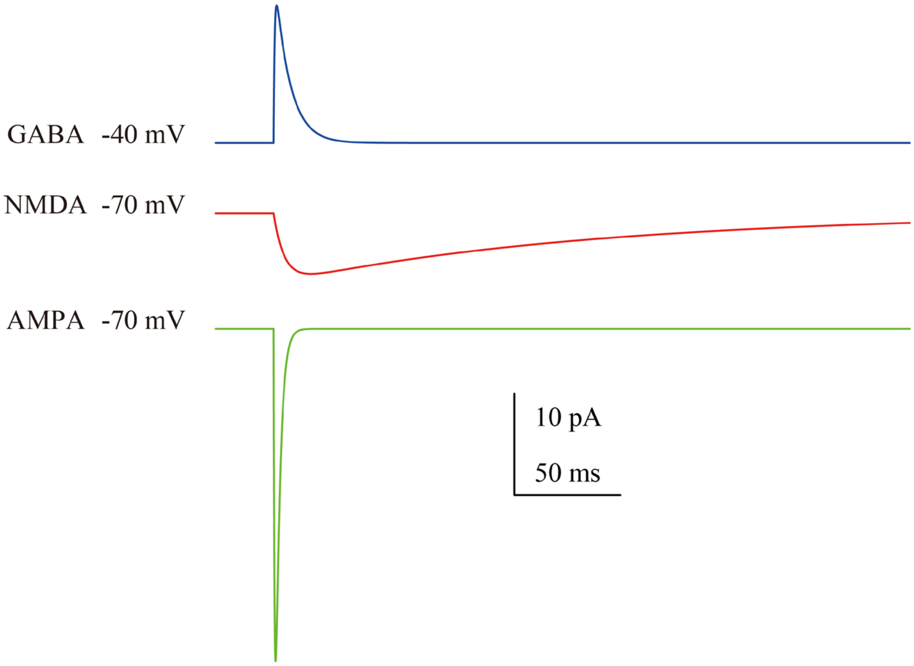

Supplementary Figure S2: The simulations of single receptor current in voltage-clamp.

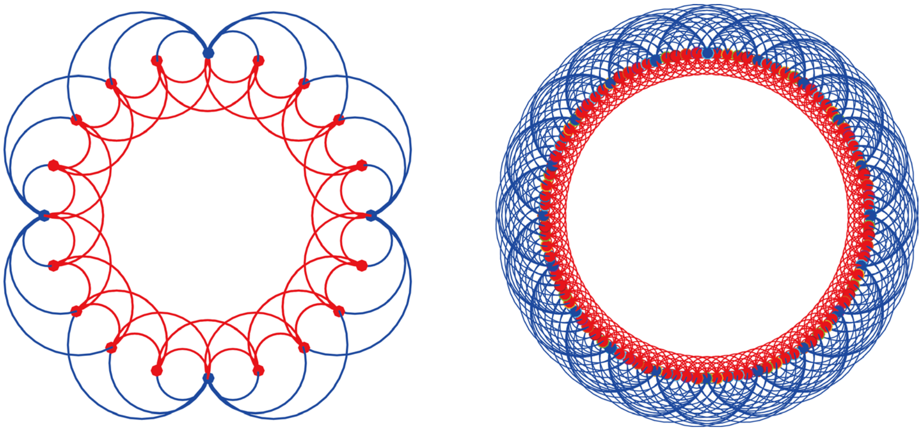

Supplementary Figure S3: The topological structures of networks. Neurons are injected in a "ring" and connected to their neighbors. The red dots are excitatory neurons and the blue dots are inhibitory GABAergic neurons. Red lines are excitatory synaptic connections from excitatory neurons to their neighbors. Blue lines are inhibitory synaptic connections from inhibitory neurons to their neighbors. Left) shows a network of twenty neurons in which sixteen of them are excitatory neurons, the others are inhibitory cells. Each excitatory neuron has four postsynaptic neighbors, and each inhibitory has six postsynaptic neighbors. Right) shows the network structure used to process the simulations. 100 neurons in which 80 of them are excitatory neurons are evenly distributed in the "ring". Each excitatory neuron has eight postsynaptic neighbors, and each inhibitory has twenty four postsynaptic neighbors.

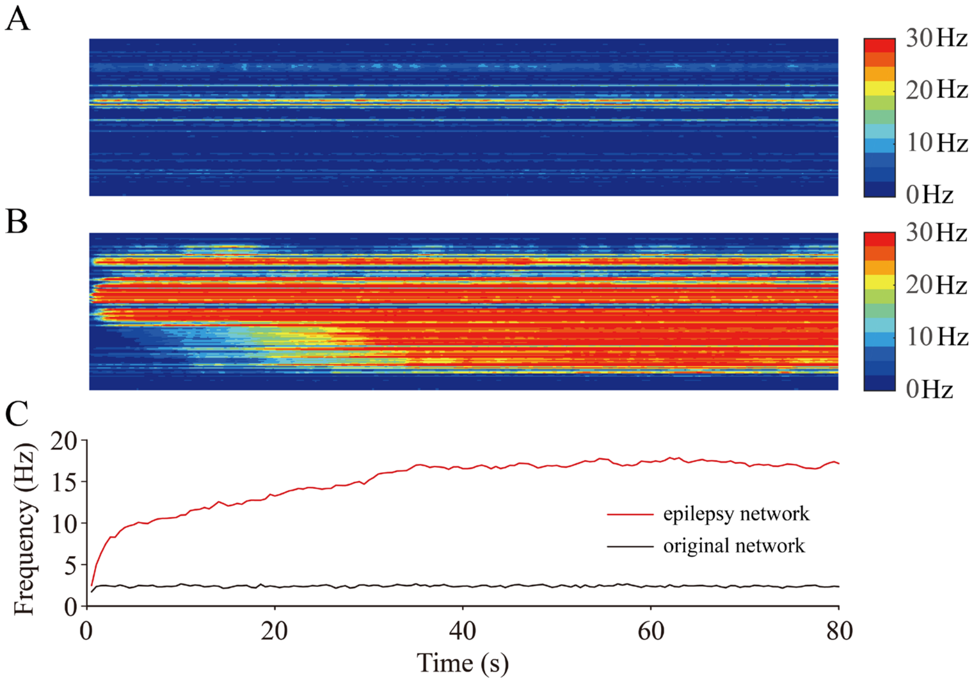

Supplementary Figure S4: The simulation of an original network and an epileptic network. A) Each neuron in the original network has no intensive fire pattern. B) Intensive spikes arise and spread in an epileptic network simulation. C) The average frequencies of excitatory neurons versus time.

Supplementary Table S1: Information of each neuron in the network

For supplementary Table S1 see Supplementary Information
